# Common Variants in the Type 2 Diabetes *KCNQ1* Gene Are Associated with Impairments in Insulin Secretion During Hyperglycaemic Glucose Clamp

**DOI:** 10.1371/journal.pone.0032148

**Published:** 2012-03-05

**Authors:** Jana V. van Vliet-Ostaptchouk, Timon W. van Haeften, Gijs W. D. Landman, Erwin Reiling, Nanne Kleefstra, Henk J. G. Bilo, Olaf H. Klungel, Anthonius de Boer, Cleo C. van Diemen, Cisca Wijmenga, H. Marike Boezen, Jacqueline M. Dekker, Esther van 't Riet, Giel Nijpels, Laura M. C. Welschen, Hata Zavrelova, Elinda J. Bruin, Clara C. Elbers, Florianne Bauer, N. Charlotte Onland-Moret, Yvonne T. van der Schouw, Diederick E. Grobbee, Annemieke M. W. Spijkerman, Daphne L. van der A, Annemarie M. Simonis-Bik, Elisabeth M. W. Eekhoff, Michaela Diamant, Mark H. H. Kramer, Dorret I. Boomsma, Eco J. de Geus, Gonneke Willemsen, P. Eline Slagboom, Marten H. Hofker, Leen M. 't Hart

**Affiliations:** 1 Molecular Genetics, Department of Pathology and Medical Biology, University Medical Center Groningen, University of Groningen, Groningen, the Netherlands; 2 Department of Endocrinology, University Medical Center Groningen, University of Groningen, Groningen, the Netherlands; 3 Department of Epidemiology, University Medical Center Groningen, University of Groningen, Groningen, the Netherlands; 4 Department of Internal Medicine, University Medical Centre Utrecht, Utrecht, the Netherlands; 5 Diabetes Centre, Isala Clinics, Zwolle, the Netherlands; 6 Department of Molecular Cell Biology, Leiden University Medical Centre, Leiden, the Netherlands; 7 Department of Internal Medicine, University Medical Center Groningen and University of Groningen, the Netherlands; 8 Medical Research Group, Langerhans, Zwolle, the Netherlands; 9 Division of Pharmacoepidemiology & Clinical Pharmacology, Utrecht Institute for Pharmaceutical Sciences, University of Utrecht, Utrecht, the Netherlands; 10 Department of Genetics, University Medical Center Groningen, University of Groningen, Groningen, the Netherlands; 11 EMGO+ Institute for Health and Care Research, VU University Medical Centre, Amsterdam, the Netherlands; 12 Department of Epidemiology and Biostatistics, VU University Medical Centre, Amsterdam, the Netherlands; 13 Department of General Practice, VU University Medical Centre, Amsterdam, the Netherlands; 14 Department of Ophthalmology, VU University Medical Center, Amsterdam, the Netherlands; 15 Department of Medical Genetics (DBG) University Medical Center Utrecht, Utrecht, the Netherlands; 16 Julius Centre for Health Sciences and Primary Care, University Medical Center Utrecht, Utrecht, the Netherlands; 17 National Institute for Public Health and the Environment (RIVM), Bilthoven, the Netherlands; 18 Diabetes Center, VU University Medical Center, Amsterdam, the Netherlands; 19 Department of Biological Psychology, VU University, Amsterdam, the Netherlands; 20 Department of Medical Statistics and Bioinformatics, section Molecular Epidemiology, Leiden University Medical Center, Leiden, the Netherlands; 21 Netherlands Consortium for Healthy Aging, the Netherlands; University of Bristol, United Kingdom

## Abstract

**Background:**

Genome-wide association studies in Japanese populations recently identified common variants in the *KCNQ1* gene to be associated with type 2 diabetes. We examined the association of these variants within *KCNQ1* with type 2 diabetes in a Dutch population, investigated their effects on insulin secretion and metabolic traits and on the risk of developing complications in type 2 diabetes patients.

**Methodology:**

The *KCNQ1* variants rs151290, rs2237892, and rs2237895 were genotyped in a total of 4620 type 2 diabetes patients and 5285 healthy controls from the Netherlands. Data on macrovascular complications, nephropathy and retinopathy were available in a subset of diabetic patients. Association between genotype and insulin secretion/action was assessed in the additional sample of 335 individuals who underwent a hyperglycaemic clamp.

**Principal Findings:**

We found that all the genotyped *KCNQ1* variants were significantly associated with type 2 diabetes in our Dutch population, and the association of rs151290 was the strongest (OR 1.20, 95% CI 1.07–1.35, *p* = 0.002). The risk C-allele of rs151290 was nominally associated with reduced first-phase glucose-stimulated insulin secretion, while the non-risk T-allele of rs2237892 was significantly correlated with increased second-phase glucose-stimulated insulin secretion (*p* = 0.025 and 0.0016, respectively). In addition, the risk C-allele of rs2237892 was associated with higher LDL and total cholesterol levels (*p* = 0.015 and 0.003, respectively). We found no evidence for an association of *KCNQ1* with diabetic complications.

**Conclusions:**

Common variants in the *KCNQ1* gene are associated with type 2 diabetes in a Dutch population, which can be explained at least in part by an effect on insulin secretion. Furthermore, our data suggest that *KCNQ1* is also associated with lipid metabolism.

## Introduction

Recent genome-wide association (GWA) studies have provided a first significant insight into the genetic architecture of type 2 diabetes, and to date, around 40 loci have been identified to be robustly associated with the disease [1]. So far, the majority of GWA scans have been performed in populations of European descent [1], The first GWA studies in East Asians have recently identified single nucleotide polymorphisms (SNPs) in a previously unreported gene, *KCNQ1*, which were associated with type 2 diabetes susceptibility [2], [3]. The original studies also confirmed this finding in populations of European descent [2], [3].

It is recognized, that in the pathophysiology of type 2 diabetes both disturbances in insulin action (liver and muscle) and in insulin secretion are early events [4] with additional factors such as age itself [5]. Therefore, it is important to examine the association between the *KCNQ1* variants and metabolic traits to elucidate the underlying diabetes-causing mechanisms.

In this study we aimed to investigate (1) the relationship of specific *KCNQ1* gene SNPs in the pathophysiology of type 2 diabetes by examining their association with metabolic traits and insulin secretion during hyperglycemic clamps, and (2) whether these SNPs relate to the risk of developing diabetes complications and to the risk of mortality among type 2 diabetes patients of Dutch origin.

## Materials and Methods

### Type 2 diabetes case-control sample description

We included 4,620 type 2 diabetes patients and 5,285 healthy controls of Dutch Caucasian origin ascertained from various study populations in the Netherlands: 1) the New Hoorn and Diabetes Care System (DCS) West-Friesland studies: 1,969 patients with type 2 diabetes and 1,951 controls with a normal glucose tolerance [6], [7], [8]; 2) the Breda study: 569 type 2 diabetes patients and 920 healthy blood bank donors [9]; 3) the Zwolle Outpatient Diabetes project Integrating Available Care (ZODIAC) study: 914 primary care patients with type 2 diabetes [10]; 4) the European Prospective Investigation Into Cancer and Nutrition-the Netherlands (EPIC-NL): 976 type 2 diabetes patients and 1,646 controls [11], [12]; 5) the Vlagtwedde/Vlaardingen cohort: 768 controls from the general population [13]; 6) the Utrecht Diabetes Epidemiology Study (UDES) study: 192 Dutch white individuals with type 2 diabetes. The ancestry in all studies except the EPIC-NL sample was determined based on self-reported information. Detailed characteristics are shown in **Table S1**.

The UDES population has not been described before: it was collected from the population-based Pharmaco-Morbidity Record Linkage System (PHARMO, www.pharmo.nl) linking drug-dispensing histories from a representative sample of Dutch community pharmacies to the national register of hospital discharges (*Landelijke Medische Registratie* (LMR)) from 1985 onwards. A retrospective cohort study of new users of blood glucose - lowering drugs (either oral hypoglycaemic agents or insulin), who were 18 years or older was designed, and 1,609 patients were recruited through community pharmacies participating in PHARMO. Diagnosis of type 2 diabetes was confirmed by self-reported information from the participants. We have checked a small sample of 24 type 2 diabetes cases and 92% of these could be confirmed according to the World Health Organisation (WHO) criteria for diagnosing type 2 diabetes. From these 1,609 patients, 255 took part in the study, returned the questionnaire that had been sent to them, and donated blood for various assessments and DNA retrieval. Laboratory measurements included plasma total cholesterol, low-density lipoprotein (LDL) cholesterol, high-density lipoprotein (HDL) cholesterol, triglycerides, fasting blood glucose, non-fasting blood glucose, and HbA_1c_. Only Dutch white individuals were included in the present study (n = 192).

All patients from studies 1–4 were diagnosed according to the WHO criteria (2-hour plasma glucose levels >11.1 mmol l^−1^ or fasting plasma glucose levels ≥7.0 mmol l^−1^).

### Data on diabetic complications and on mortality in type 2 diabetes patients

The presence of macrovascular complications (n = 284), nephropathy (n = 442), neuropathy (n = 333), and diabetic retinopathy (n = 465) was available in a subset of type 2 diabetes patients from DCS West-Friesland, the ZODIAC and the EPIC-NL studies (**Table S2**). [8], [10], [12]. A diagnosis of diabetic retinopathy in the DCS West-Friesland was based on the examination of fundus photography graded by an ophthalmologist according to the EURODIAB Study grading system as described elsewhere [8]. A diagnosis of diabetic retinopathy in the ZODIAC cohort was based on an ophthalmologist's examination that demonstrated microaneurysms, macular edema, or (pre-) proliferative retinopathy. In the EPIC-NL study data on diabetic complications was collected during the ascertainment and verification of prevalent and incident diabetes cases in the Dutch contributor to the EPIC cohort. Diabetes was ascertained via self-report, linkage to registers of hospital discharge diagnoses and a urinary glucose strip test. Ascertained diabetes cases were verified against general practitioner (GP) or pharmacist information using mailed questionnaires [12]. The GP questionnaire contained 12 questions on diabetes. The questions on in what year and which type of diabetes had been diagnosed, on how the diagnosis was established, on treatment during the first year after diagnosis and current treatment (diet, oral glucose lowering medication, insulin), and whether the patient suffered from long-term complications were asked. When the GP was unknown, pharmacist information was used to verify the diagnosis of diabetes. The pharmacist questionnaire contained eight questions concerning use of diabetes medication [12].

Data from the ZODIAC study on mortality were collected after a follow-up period of 10 years in a prospective Zwolle cohort of type 2 diabetes patients by retrieving life status and cause of death from records maintained by the hospital and the GPs [10].

### Hyperglycaemic clamp study

Participants from three independent studies from the Netherlands were used: 138 impaired glucose tolerance (IGT) subjects from the Hoorn study, 74 subjects (63 normal glucose tolerance (NGT)/11 IGT) from the Utrecht clamp cohort and 123 twins and sibs (116 NGT/7 IGT) from the Netherlands Twin Register (NTR) [14], [15]. The latter cohort is a family based twin study, which includes 66 monozygotic, and 28 dizygotic twins as well as 29 of their nontwin sibs recruited from 50 families [15]. The clinical characteristics of the study groups are given in **Table S3**. Details of these study groups and the clamp procedure have previously been described elsewhere [14], [15].

All participants (n = 335) underwent a hyperglycaemic clamp at 10 mmol/l glucose for at least 2 h. First-phase insulin secretion was determined as the sum of the insulin levels during the first 10 min of the clamp. Second-phase insulin secretion was determined as the mean of the insulin levels during the last 40 min of the second hour of the clamp (80–120 min). The insulin sensitivity index (ISI) was calculated by relating the glucose infusion rate (M) to the plasma insulin concentration (I) during the last 40 min of the second hour of the clamp (M/I). The disposition index (DI) was calculated by multiplication of first-phase insulin secretion and ISI in order to quantify insulin secretion in relation to the ambient insulin sensitivity [15].

All study protocols were approved by local institutional review boards or hospital medical ethics committees (the New Hoorn and DCS West-Friesland studies were approved by the medical ethics committee of the VU University Medical Center Amsterdam; the Breda, EPIC-NL, and UDES studies were approved by the Medical Ethics Committee of the University Medical Center Utrecht; the ZODIAC study was approved by the local Medical Ethical Committee of the Isala Clinics; the Vlagtwedde/Vlaardingen study was approved by the Medical Ethics Committee of the University Medical Center Groningen, the ‘clamp studies’ were approved by the medical ethics committees of the VU University Medical Center Amsterdam and the University Medical Center Utrecht). All participants gave their written informed consent.

### Genotyping

Based on the original GWA scans and the replication studies in Europeans, we selected gene variants with a minor allele frequency (MAF) >5% - rs151290, rs2237892, and rs2237895 in the *KCNQ1*, which were reported to be strongly associated with type 2 diabetes in European population [2], [3], [16], [17], [18]. The two SNPs rs2237895 and rs2237892 showed the strongest association with T2D and were replicated in European population in the original studies by Unoki et al. and Yasuda et al., respectively [2], [3]. The variant rs151290 showed strong association in the third screening in the original study by Yasuda et al. [3], and was also reported to be associated with insulin secretion in a German population by Mussig et al. [18].

These variants were genotyped in all the samples except the EPIC-NL study samples using Taqman assays (Applied Biosystems, Applied Biosystems, Nieuwerkerk a/d IJssel, The Netherlands) and were analyzed using a TaqMan 7900HT (Applied Biosystems). The DNA samples were processed in 384-well plates. Each plate contained 16 genotyping controls (4 duplicates of 4 different Centre d'Etude du Polymorphisme Humain (CEPH) samples). There were no discordances in the genotypes of any of the CEPH samples and the CEU data available from HapMap. The genotype success rate was 95.6%, 97.6% and 98.2% for rs151290, rs2237892, and rs2237895, respectively. For the individuals from the EPIC-NL study, the genotypes data for the two SNPs - rs2237892 and rs2237895, were available. The EPIC-NL samples were genotyped using the Illumina IBC v.3 array (also referred to as the CardioChip or the Human Cardiovascular Disease [HumanCVD] BeadChip [Illumina] [19]). The genotyping information was not available for rs151290 because this SNP was not included on the CardioChip. The rs2237892 and rs2237895 SNPs were clustered into genotypes with the use of the Illumina Beadstudio software and were subjected to quality control filters at the sample (i.e. only samples with call rate >95% and only not related and individuals of European ancestry were included) and SNP levels (i.e. SNPs with a call rate <95% or HWE p<10^−6^ were removed).

### Statistical analysis

The genotype frequencies were tested for Hardy-Weinberg equilibrium (HWE) by χ^2^ analysis. In the genotyped samples from the EPIC-NL study, pi-hat, a measure of identity by descent, was calculated to exclude cryptic relatedness and duplicate samples (pi-hat>0.2) via the method implemented in PLINK [20]; EIGENSTRAT method was used to compute principal component with HapMap panels as reference standards to exclude the population outliers [21].

To test for association of genotypes and type 2 diabetes and its complications, genotype-based odds ratios (OR) with 95% confidence intervals (CI) were calculated in the combined sample of type 2 diabetes patients and controls using a logistic regression model, with individuals homozygous for the non-risk allele as the reference group. The risk and non-risk alleles were defined based on the previous reports [2], [3], [16], [17], [18]. The association between genotypes and metabolic traits (BMI, HbA1c, fasting glucose, HDL- and LDL cholesterol, total cholesterol, triglyceride) was determined using linear regression analysis. This analysis was restricted to control individuals to avoid diabetes status or treatment masking potential effects of the variants on these phenotypic traits. All analyses were done under the additive model and the presented p-values are adjusted for age, sex and study center, with the continuous traits log-transformed prior to statistical comparisons. The effect of the risk alleles on the responses during hyperglycaemic clamping was examined by calculating the β values for the risk allele with linear generalized estimating equations, which takes into account the family relatedness when computing the standard errors (i.e., in the twin sample from the Netherlands Twin Register study). Only for rs2237892 the non-risk allele was used for the latter calculations since our sample did not have any homozygotes for the risk allele. All outcome variables were log-transformed prior to analysis. Analyses of hyperglycaemic clamp data were also adjusted for age, sex, BMI, study center and glucose tolerance status. A Cox proportional hazard model was used to assess association between the SNPs and total mortality with correction for age and sex.

To account for the number of independent tests, a *p-*value of <0.0033 (α = 0.05/15) was considered statistically significant, given independent tests for 15 outcomes (type 2 diabetes status, its complications (i.e. retinopathy, nephropathy, neuropathy), metabolic traits (i.e. (BMI, HbA1c, fasting glucose, HDL, LDL, total cholesterol, triglyceride) and the parameters related with insulin secretion). However, as this level is probably too stringent as a Bonferroni correction assumes independency of the tests, which is clearly not the case in this study, *p*-values between 0.05 and 0.0033 were considered nominally significant. All statistical analyses were performed using the SPSS program, version 14.0 for Windows (SPSS, Chicago, IL, USA).

Power calculation was performed using Quanto software (http://hydra.usc.edu/gxe/). Assuming a disease prevalence in the Dutch population of 0.04 [6], our study had more than 95% power to detect the ORs of 1.30, 1.29 and 1.24 reported by Unoki et al. and Yasuda et al. for rs151290, rs2237892, and rs2237895, respectively, with a significance level of 0.05 for the allele frequencies of the risk allele of 0.79, 0.93, and 0.40, and assuming a log-additive model [2], [3].

## Results

### Effect of the KCNQ1 polymorphisms on type 2 diabetes risk in Dutch population

We genotyped the *KCNQ1* variants rs151290, rs2237892, and rs2237895 in a total of 4,620 type 2 diabetes patients and 5,285 healthy controls. Baseline characteristics of the study population are shown in **Table 1**. The controls were in Hardy-Weinberg equilibrium (HWE) (χ^2^ = 0.33, *p* = 0.57 for rs151290, χ^2^ = 3.21, *p* = 0.07 for rs2237892, and χ^2^ = 0.09, *p* = 0.77 for rs2237895). Genotype and allele frequencies in both controls and type 2 diabetes cases are summarised in **Table 2**. In the control group the observed MAF were 24%, 5%, and 41%, for rs151290, rs2237892, and rs2237895, respectively, which are similar to the MAF in the European population reported previously [2], [3], [18]. In our sample, all three variants were associated with type 2 diabetes. The risk alleles were identical to those in previous studies: the major C-allele for rs151290 and for rs2237892, the minor C-allele for rs2237895 [2], [3], [16], [17], [18]. The variant rs151290 showed the strongest association with the disease (OR 1.20, 95% CI 1.07–1.35, *p* = 0.002, adjusted for age, sex and study center).

**Table 1:** Clinical characteristics of the study participants.

Trait	T2D patients (n = 4620)		Controls (n = 5285)	
	n	Mean ± SD	n	Mean ± SD
Male (%)	4616	1999 (43.3)	5272	2209 (41.9
Age-at-study (years)	4617	64.3±10.6	5266	51.1±10.1
Age at diagnosis (years)	3497	58.7±11.6	-	-
BMI (kg/m2)	4550	29.4±4.9	4349	26.0±3.8
HbA1c (%)	4370	7.1±1.3	3565	5.4±0.4
Fasting glucose	2496	8.3±2.3	1951	5.3±0.4
HDL-cholesterol (mmol/l)	4187	1.2±0.3	3553	1.4±0.4
LDL-cholesterol (mmol/l)	2746	2.8±0.9	3545	3.2±0.9
Total cholesterol (mmol/l)	4226	5.1±1.2	3571	5.4±1.0
Triglyceride (mmol/l)	4232	2.0±1.3	3564	1.4±0.9

The data are presented as mean ± SD. BMI: Body Mass Index. HbA_1c_: haemoglobin A_1c_. HDL: high density lipoprotein. LDL: low density lipoprotein. T2D: type 2 diabetes.

**Table 2:** Association of the *KCNQ1* variants with type 2 diabetes in the Dutch population.

SNP	Allele data				Genotype distribution (%)			*p*-value^a^^,^ b
Group	Major/minor allele	RAF	OR (95% CI)	*p*-value^a^	11	12	22	
**rs151290**								
T2D	**C**/A	0.78	1.20 (1.07–1.35)	**0.002**	2081 (60.2)	1220 (35.3)	153 (4.4)	**0.003**
Control		0.76			2045 (58.3)	1258 (35.9)	204 (5.8)	
**rs2237892**								
T2D	**C**/T	0.96	1.16 (0.97–1.40)	0.11	4149 (92.0)	348 (7.7)	14 (0.3)	**0.002**
Control		0.95			4638 (90.0)	507 (9.8)	7 (0.1)	
**rs2237895**								
T2D	A/**C**	0.43	1.09 (1.00–1.17)	0.035	1522 (33.5)	2158 (47.4)	869 (19.1)	**0.006**
Control		0.41			1803 (34.8)	2516 (48.6)	863 (16.7)	

a Adjusted for age, sex and study center.

b p-value for the additive model.

For each variant the C-allele is the risk allele for type 2 diabetes as identified by previous studies. RAF: risk allele frequency. T2D: type 2 diabetes.

### Effect of KCNQ1 polymorphisms on insulin secretion as assessed with hyperglycaemic glucose clamps

To investigate potential mechanisms by which the variants in *KCNQ1* may contribute to type 2 diabetes susceptibility, we used regression analysis to examine the effects of *KCNQ1* genotypes on first- and second-phase of glucose stimulated insulin secretion, insulin sensitivity index (ISI) and disposition index (DI) in a sample of non-diabetic individuals in whom hyperglycaemic clamp was performed (**Table 3**). We found nominal association between the C-allele of rs151290 and decreased insulin secretion during first-phase and increased ISI (*p* = 0.025 and *p* = 0.006, adjusted for age, sex, study center, BMI and glucose tolerance status, respectively) as well as nominal relationship between the C-allele of rs2237895 and higher ISI values (*p* = 0.034, adjusted for age, sex, study center, BMI and glucose tolerance status). In addition, we observed that carriers of the non-risk allele for rs2237892 had significantly higher second phase insulin secretion and nominally significant lower ISI compared to the homozygotes for the C-risk allele (*p* = 0.0016 and *p* = 0.036, adjusted for age, sex, study center, BMI and glucose tolerance status, respectively). None of the *KCNQ1* variants had an effect on the DI in our study.

**Table 3:** Effect of *KCNQ1* variants rs151290, rs2237892 and rs2237895 on beta-cell function as assessed with hyperglycaemic clamp.

SNP	Genotype (N)		1st phase insulin response (pmol/l)	2nd phase insulin response (pmol/l)	ISI (µmol • min^−1^ • kg^−1^ • pmol/l^−1^)	DI (µmol • min^−1^ • kg^−1^)
**rs151290**						
	AA (19)					
	**C**A (90)	β (sem)^a^	−0.046 (0.020)	−0.029 (0.021)	+0.059 (0.021)	+0.011 (0.022)
	**CC** (226)	*p-*value^a^	**0.025**	0.16	**0.0061**	0.63
**rs2237892**						
	TT (0)					
	**C**T (28)	β (sem)^a^	+0.052 (0.036)	+0.126 (0.040)	−0.091 (0.044)	−0.028 (0.039)
	**CC** (301)	*p-*value^a^	0.15	**0.0016**	**0.036**	0.47
**rs2237895**						
	AA (86)					
	A**C** (180)	β (sem)^a^	−0.024 (0.021)	−0.029 (0.022)	+0.047 (0.022)	+0.026 (0.019)
	**CC** (63)	*p-*value^a^	0.26	0.19	**0.034**	0.18

a Adjusted for glucose tolerance status (NGT/IGT), study center, age, gender and BMI.

All variables were log-transformed before analysis. *p*-values were computed for different additive models using linear generalized estimating equations (GEE) which takes into account the family relatedness when computing the standard errors. Alleles in bold are the risk alleles for type 2 diabetes identified by previous studies.

DI, disposition index; IGT, impaired glucose tolerance; ISI, insulin sensitivity index; ND, not determined; NGT, normal glucose tolerance.

### Relationship of KCNQ1 polymorphisms with metabolic parameters

Next, we investigated whether the *KCNQ1* polymorphisms influenced relevant clinical parameters, such as BMI, HbA_1c_, fasting glucose, HDL, LDL, total cholesterol and triglycerides, in non-diabetic individuals (**Table 4**). Under an additive genetic model, the diabetes risk allele of rs2237892 was nominally associated with increased LDL level and significantly associated with a higher level of total cholesterol (*p* = 0.015 and *p* = 0.003, adjusted for age, sex and study center, respectively). In addition, carriers of the C-risk allele of rs2237895 had nominally significantly higher HbA_1c_ levels (*p* = 0.036, adjusted for age, sex and study center).

**Table 4:** Effect of *KCNQ1* variants rs151290, rs2237892 and rs2237895 on quantitative metabolic traits in non-diabetic individuals.

	rs151290			*p*-value^a^^,^ b
Genotype (N)	AA (204)	A**C** (1252)	**CC** (2035)	
BMI (kg/m^2^)	26.5±3.6	26.0±3.8	26.3±3.9	0.20
HbA1c (%)	5.30±0.23	5.33±0.26	5.33±0.27	0.52
Fasting glucose (mmol/l)	5.31±0.36	5.31±0.38	5.30±0.38	0.82
HDL cholesterol (mmol/l)	1.62±0.40	1.56±0.44	1.55±0.41	0.07
LDL cholesterol (mmol/l)	3.24±0.83	3.31±0.87	3.31±0.87	0.65
Total cholesterol (mmol/l)	5.44±0.93	5.46±0.95	5.44±0.98	0.76
Triglyceride (mmol/l)	1.29±0.60	1.30±0.69	1.31±0.73	0.85

	rs2237892			
Genotype (N)	TT (7)	**C**T (507)	**CC** (4623)	
BMI (kg/m^2^)	23.5±3.2	26.1±3.9	26.0±3.9	0.46
HbA1c (%)	5.18±0.14	5.40±0.51	5.39±0.39	0.71
Fasting glucose (mmol/l)	5.07±0.06	5.27±0.40	5.31±0.37	0.29
HDL cholesterol (mmol/l)	1.33±0.29	1.41±0.40	1.43±0.41	0.24
LDL cholesterol (mmol/l)	3.00±0.84	3.08±0.80	3.23±0.89	**0.015**
Total cholesterol (mmol/l)	5.11±0.92	5.22±0.96	5.40±1.02	**0.003**
Triglyceride (mmol/l)	1.57±0.70	1.39±0.81	1.42±0.86	0.79

	rs2237895			
Genotype (N)	AA (1797)	A**C** (2509)	**CC** (857)	
BMI (kg/m^2^)	26.0±3.8	25.9±3.9	26.1±3.8	0.99
HbA1c (%)	5.37±0.40	5.40±0.40	5.41±0.41	**0.036**
Fasting glucose (mmol/l)	5.30±0.37	5.31±0.38	5.29±0.36	0.90
HDL cholesterol (mmol/l)	1.44±0.41	1.42±0.42	1.42±0.40	0.82
LDL cholesterol (mmol/l)	3.20±0.87	3.21±0.87	3.25±0.91	0.21
Total cholesterol (mmol/l)	5.36±1.00	5.38±1.00	5.45±1.07	0.14
Triglyceride (mmol/l)	1.39±0.86	1.41±0.81	1.48±0.96	0.17

a Adjusted for age, sex and study center.

b p-value for the additive model.

The data are presented as mean±SD. All variables were log-transformed before analysis. Alleles in bold are the risk alleles for type 2 diabetes identified by previous studies. BMI: Body Mass Index. HbA_1c_: haemoglobin A_1c_ (glucose bound to haemoglobin). HDL: high density lipoprotein. LDL: low density lipoprotein.

### Relationship of KCNQ1 polymorphisms with diabetes complications and mortality

We further investigated whether there was an association between the *KCNQ1* gene SNPs and various type 2 diabetes complications and mortality. We did not observe any association of *KCNQ1* SNPs with diabetic complications, although there was a trend of the homozygous carriers of the risk C-allele of rs2237895 being more frequent among the diabetic patients with retinopathy compared to the patients without it (22.5 vs. 19.2%, *p* = 0.06, adjusted for age, sex, study and duration of type 2 diabetes).

Data on mortality were collected after a follow-up period of 10 years in the ZODIAC study; the characteristics of the study population are shown in **Table S4**. After a median follow-up period of 9.5 years, a total of 358 (39%) patients had died, of these 146 (41%) had died from cardiovascular disease and 82 (23%) deaths were cancer-related. The cause of death was known for 336 (97.6%) patients. All the baseline characteristics - age at baseline, gender, duration of diabetes, smoking status, BMI, systolic blood pressure, HbA1c, serum creatinine, total cholesterol to HDL-cholesterol ratio, and albuminuria creatinine ratio – were not significantly different in the groups according to the *KCNQ1* SNPs. In our study, there was no evidence of association for the *KCNQ1* SNPs with mortality. The age and sex adjusted HR for patients carrying one or both risk alleles compared to non-carriers were HR 0.97 (95% CI 0.56–1.66, *p* -value 0.91) and HR 0.94 (95% CI 0.75–1.18, *p* –value 0.56); HR 0.98 (95% CI 0.14–7.03, *p* -value 0.97) and HR 1.10 (95% CI 0.74–1.64, *p* -value 0.64); HR 1.10 (95% CI 0.81–1.49, *p* -value 0.32) and HR 0.92 (95% CI 0.68–1.23, *p* -value 0.56) for rs151290, rs2237892, rs2237895, respectively.

## Discussion

Two independent GWA studies recently performed in Japanese populations identified *KCNQ1* as a type 2 diabetes susceptibility gene [2], [3]. We here confirm the association of the *KCNQ1* common variants with an increased risk of type 2 diabetes in a Dutch population. The individuals carrying the same at-risk alleles *C*, as reported in the Japanese studies [2], [3], had a modestly increased risk of developing type 2 diabetes, with a population attributable risk from 0.6% to 4.3%. These results are also consistent with previous studies performed in Caucasian populations [2], [3], [16], [17], [22], [23]. In the present study, we demonstrate in a large cohort of subjects having undergone hyperglycaemic glucose clamps that the risk allele of the *KCNQ1* SNP is significantly associated with reduced glucose-stimulated second-phase insulin secretion. In addition, we report a significant association of *KCNQ1* variants with impaired lipid parameters. We could not find any significant relationship of the risk alleles of the *KCNQ1* gene with type 2 diabetes complications or mortality.

The variants rs151290, rs2237892, and rs2237895 are located in intron 15 of the *KCNQ1* gene on chromosome 11p15, encoding the pore-forming alpha subunit of the I(_Ks_) channel, a voltage-gated potassium channel that is expressed in a number of tissues, notably, the heart, pancreas, kidneys and intestine [2], [3]. The encoded protein plays a role in the electrical depolarisation of the cell membrane in the heart and presumably in pancreas beta cells. It is likely to be involved in insulin secretion, although there are other possibilities, such as secretory processes in incretin (GLP-1 and/or GIP) -producing cells [2], [3]. Our results provide compelling evidence that the *KCNQ1* rs2237892 variant is associated with impaired second-phase insulin secretion. Moreover, these data confirm the observations from the previous studies in which the relationship between the type 2 diabetes risk alleles in *KCNQ1* and reduced levels of various measures of insulin secretion have been reported [17], [18], [22], [24], and hence, supports the hypothesis on a potential link between *KCNQ1* and impaired beta-cell function [2], [3], [17], [18], [22], [23], [24], [25], [26].

The importance of the investigation of the various aspects of insulin secretion was highlighted previously [27]. It is thought that first phase insulin secretion is reflecting the rapid recruitment and release of insulin granules from the readily releasable pool, while second phase is due to the release of granules located further away from release site (reserve pool) and due to (new) insulin synthesis [28]. In the present study, we used the hyperglycaemic clamp technique, generally considered to be the gold standard for quantification of first- and second-phase insulin secretion to measure beta-cell function [29]. We show, for the first time, an association between a variant in the *KCNQ1* gene (rs2237892) *and* second-phase insulin secretion. This observation indicates that *KCNQ1* might play a role in second-phase insulin secretion suggesting a novel potential link between *KCNQ1* and impaired beta-cell function via the decreased release of newly formed insulin following glucose stimulation. It is worth noting, that it appears from previous studies from others and ourselves that the majority of the known beta-cell loci influence mainly the first-phase insulin secretion [17], [18], [30], [31], [32]. Although we did not detect a significant effect of *KCNQ1* on first-phase insulin secretion, we observed decreased insulin secretion among the C-allele carriers of rs151290, which is in agreement with the recent study by Mussing et al. [18]. Also, Holmkvist et al. reported the association between the *KCNQ1* gene and reduced estimates of first-phase insulin release [17]. Both above-mentioned studies used data obtained during oral glucose tolerance test [17], [18]. Our data derived from the hyperglycaemic clamp extends these finding and suggests that *KCNQ1* may have an effect regarding second phase secretion and therefore may have additional effects apart from early, rapid recruitment and exocytosis of insulin granules after glucose stimulation. In addition, there was a surprising trend of the association between all three risk alleles and higher ISI values. Interestingly, Boini et al. have recently shown that insulin sensitivity is strikingly increased in the *KCNQ1* knockout mouse, which agrees with our results [33]. Thus, although the *KCNQ1* gene is primarily a candidate gene for impaired insulin secretion, its effect on tissue-specific insulin-sensitivity independent of changes in insulin secretion is plausible.

In addition, we found a significant association between the type 2 diabetes risk allele of rs2237892 and higher level of total cholesterol and a trend towards increased level of LDL among the carriers of the same allele. Also, we observed a lower level of HDL in the carriers of the type 2 diabetes risk allele of rs151290 variant than in non-carriers. Interestingly, Chen et al. have recently reported the relationship of *KCNQ1* variants with higher level of triglycerides (i.e. also rs2237892) and lower levels of HDL [34]. Although the mechanisms behind the observed associations are not clear, taken together, these data suggest that *KCNQ1* may have an effect on lipid metabolism. Further studies are needed to elucidate the underlying molecular mechanisms.

In the present study, we found no significant evidence for an association of *KCNQ1* with diabetic complications or mortality in type 2 diabetes patients. Recently, Ohshige et al. reported that *KCNQ1* might be a potential susceptibility gene for diabetic nephropathy in a Japanese population [35]. In our sample, we could not confirm that finding. These results could be due to the relatively small sample of the patients with nephropathy in our study (there were 442 patients in our study versus 1545 diabetic patients with overt nephropathy in the study by Ohshige et al. [35]). Next, the association with nephropathy was not easy to detect due to differences in risk-allele frequency (33% in Japanese versus 5% in European) of the rs2237897 variant associated with nephropathy (r^2^ = 0.6 with rs2237892) [35]. Our study had 20% power at *p* = 0.05 to detect the OR of 1.22 reported by Ohshige et al. [35] assuming prevalence of nephropathy of 0.40 among type 2 diabetes patients [36] and an additive model. In addition, we cannot exclude a proportion of patients with diabetic nephropathy in the reference sample. Therefore, additional replication attempts in larger studies with detailed information on the complications status are warranted.

It needs to be noted that in the current study we used control subjects younger than the patients. Thus, we cannot exclude that some of these individuals may develop diabetes in later life. However, this would result in slight reduction in statistical power and also in the ORs and would lead to the underestimation of the “true” effects of the *KCNQ1* variants on susceptibility to T2D in our study. Next, the individuals in the hyperglycaemic clamp study were younger than the type 2 diabetes patients used in the association analysis. Also, the mean age of the participants in the groups of the clamp study was different. To test the effect of age on the relationship between the *KCNQ1* variants and insulin secretion, we performed an additional analysis in the different clamp study groups. The results of these analyses were similar to the observations in the whole clamp sample (**Table S5**). Another limitation of the current study that needs to be taken into account is the lack of correction for population stratification. Since the ethnicity was determined based on self-reported information (except for the EPIC-NL study), we cannot rule out presence of the participants of non-Dutch Caucasian origin in our study. However, as the same alleles are the risk alleles for type 2 diabetes in populations of different ancestries (the minor allele for rs2237895, the major allele for both rs151290 and rs2237892) [2], [3], and the non-Caucasian participants are very likely to be present in both control and patients groups, that will have had only a minor effect on the results in our study. In addition, the MAFs in our study were similar to the MAFs reported in European populations [2], [3], [16], [17], [18].

Finally, it is still unclear whether the variants rs151290, rs2237892, and rs2237895 located in intron 15 of the *KCNQ1* gene directly affect the gene expression or are in strong linkage disequilibrium with a causal polymorphism in *KCNQ1* or a neighbouring gene. Also, it was recently shown that one of the type 2 diabetes association signals maps to a part of the *KCNQ1* sequence which also encodes a different transcript (KCNQ1OT1) which is known to be an key regulator of other genes in the region, including *KCNQ1* itself, but also *CDKN1C*, a gene already heavily implicated in islet development [23].

In conclusion, we here show that common variation in the *KCNQ1* gene affect second-phase insulin secretion and confirm the association of the gene with type 2 diabetes in a Dutch population. We also found that the variants in the *KCNQ1* gene may have an effect on lipid metabolism. These results provide new insight into the complex pathogenesis of diabetes.

## Supporting Information

Table S1 **Detailed clinical characteristics of the study participants from different studies.** The data are presented as mean±SD. BMI: Body Mass Index. HbA_1c_: haemoglobin A_1c_ (glucose bound to haemoglobin). HDL: high density lipoprotein. T2D: type 2 diabetes. NA: not applicable.
(DOC)

Table S2 **Data on diabetic complications in type 2 diabetes patients from the DCS West-Friesland, the ZODIAC and the EPIC-NL studies.**
(DOC)

Table S3 **Clinical characteristics of the individual hyperglycaemic clamp study samples.** Data are represented as means ± SD or median (interquartile range). NTR = Netherlands Twin Register.
(DOC)

Table S4 **The characteristics of the Zwolle study population.** Data are presented as mean ±SD or median with interquartile range for non-normally distributed data or %.* P<0.05, ** P<0.01, ***P<0.001, tested with Student's t-test or Mann-Whitney U as appropriate.
(DOC)

Table S5 **Effect of** ***KCNQ1*** **variants rs151290, rs2237892 and rs2237895 on beta-cell function as assessed with hyperglycaemic clamp.** Analysis was adjusted for glucose tolerance status (NGT/IGT), study center, age, gender and BMI. All variables were log-transformed before analysis. *p*-values were computed for different additive models using linear generalized estimating equations (GEE) which takes into account the family relatedness when computing the standard errors. Alleles in bold are the risk alleles for type 2 diabetes identified by previous studies. DI, disposition index; IGT, impaired glucose tolerance; ISI, insulin sensitivity index; ND, not determined; NGT, normal glucose tolerance.
(DOC)
