# Correction: Common Variants in the Type 2 Diabetes *KCNQ1* Gene Are Associated with Impairments in Insulin Secretion During Hyperglycaemic Glucose Clamp

**DOI:** 10.1371/annotation/d44162c6-c882-4f98-b355-4feedd1a8f46

**Published:** 2012-06-13

**Authors:** Jana V. van Vliet-Ostaptchouk, Timon W. van Haeften, Gijs W. D. Landman, Erwin Reiling, Nanne Kleefstra, Henk J. G. Bilo, Olaf H. Klungel, Anthonius de Boer, Cleo C. van Diemen, Cisca Wijmenga, H. Marike Boezen, Jacqueline M. Dekker, Esther van 't Riet, Giel Nijpels, Laura M. C. Welschen, Hata Zavrelova, Elinda J. Bruin, Clara C. Elbers, Florianne Bauer, N. Charlotte Onland-Moret, Yvonne T. van der Schouw, Diederick E. Grobbee, Annemieke M. W. Spijkerman, Daphne L. van der A, Annemarie M. Simonis-Bik, Elisabeth M. W. Eekhoff, Michaela Diamant, Mark H. H. Kramer, Dorret I. Boomsma, Eco J. de Geus, Gonneke Willemsen, P. Eline Slagboom, Marten H. Hofker, Leen M. 't Hart

There was an error in the affiliations of the last author. Leen M. 't Hart is not affiliated with affiliation 5. The Correct affiliations of Leen M. 't Hart are:

6 Department of Molecular Cell Biology, Leiden University Medical Centre, Leiden, the Netherlands,

20 Department of Medical Statistics and Bioinformatics, section Molecular Epidemiology, Leiden University Medical Center, Leiden, the Netherlands. 

