# Supplementary material for: Common Variants in the Type 2 Diabetes KCNQ1 Gene Are Associated with Impairments in Insulin Secretion During Hyperglycaemic Glucose Clamp
Source: PLoS One. 2012 Mar 5;7(3):e32148. doi: 10.1371/journal.pone.0032148 (PMC3293880; doi:10.1371/journal.pone.0032148)
Supplement: Table S1 — Detailed clinical characteristics of the study participants from different studies. The data are presented as mean±SD. BMI: Body Mass Index. HbA1c: haemoglobin A1c (glucose bound to haemoglobin). HDL: high density lipoprotein. T2D: type 2 diabetes. NA: not applicable. (DOC) [file pone.0032148.s001.doc]

**Supplementary table 1.** Detailed clinical characteristics of the study participants from different studies.

| Trait | Type 2 diabetes patients | | | | | | Controls | | | |
| --- | --- | --- | --- | --- | --- | --- | --- | --- | --- | --- |
|  | n | Breda (n=569) | n | UDES (n=192) | n | Zodiac(n=914) | n | Blood bank controls (n=920) | n | Vlagtwedde (n=768) |
| Male/female (%) | 567 | 255 (45.0) / 312 (55.0) | 190 | 103 (54.2) / 87 (45.8) | 914 | 386 (42.2) / 528 (57.8) | 911 | 557 (61.1) / 354 (38.9) | 764 | 409 (53.5) / 355 (46.5) |
| Age-at-study (years) | 569 | 69.9±9.9 | 189 | 65.2±10.7 | 914 | 67.4±11.3 | 905 | 47.8±12.7 | 764 | 51.2±9.3 |
| Age at diagnosis (years) | 557 | 62.6±11.1 | 189 | 58.3±11.3 | 912 | 59.8±±12.4 |  | – |  | – |
| BMI (kg/m^2^) | 564 | 28.0±4.2 | 182 | 28.8±4.7 | 912 | 28.9±4.6 |  | NA | 764 | 27.8±4.0 |
| HbA_1c_ (%) | 506 | 7.3±1.1 | 180 | 7.1±0.9 | 914 | 7.3±1.1 |  | NA |  | NA |
| HDL-cholesterol (mmol/l) | 517 | 1.2±0.3 | 177 | 1.2±0.3 | 914 | 1.2±0.3 |  | NA |  | NA |
| Total cholesterol (mmol/l) | 517 | 5.3±1.0 | 185 | 5.6±1.2 | 914 | 5.6±1.1 |  | NA |  | NA |
| Triglyceride (mmol/l) | 517 | 1.9±1.0 | 177 | 2.4±1.7 | 914 | 2.5±1.5 |  | NA |  | NA |

The data are presented as mean±SD. BMI: Body Mass Index. HbA_1c_: haemoglobin A_1c_ (glucose bound to haemoglobin). HDL: high density lipoprotein. T2D: type 2 diabetes. NA: not applicable

| Trait | n | New Hoorn / DCS West-Friesland T2D (n=1.969) | n | New Hoorn / DCS West-Friesland controls (n=1.951) | n | EPIC-NL T2D (n=976) | n | EPIC-NL controls (n=1.646) |
| --- | --- | --- | --- | --- | --- | --- | --- | --- |
| Male/female (%) | 1969 | 1083 (55.0) / 886 (45.0) | 1951 | 871 (44.6) / 1080 (55.4) | 976 | 172 (17.6) / 804 (82.4) | 1646 | 372 (22.6) / 1274 (77.4) |
| Age-at-study (years) | 1969 | 64.1 ± 10.5 | 1951 | 53.1 ± 6.7 | 976 | 58.2 ± 6.9 | 1646 | 50.6 ± 117 |
| Age-at-diagnosis (years) | 1839 | 57.0 ± 11.0 |  | – |  | ± | – | – |
| BMI (kg/m^2^) | 1917 | 30.0 ± 5.3 | 1940 | 25.6 ± 3.6 | 975 | 29.7 ± 4.6 | 1645 | 25.6 ± 3.8 |
| HbA_1c_ (%) | 1809 | 6.9 ± 1.1 | 1950 | 5.3 ± 0.3 | 961 | 7.2 ± 1.7 | 1615 | 5.5 ± 0.5 |
| Fasting blood glucose (mmol/l) | 1808 | 8.2 ± 2.2 | 1951 | 5.3 ± 0.4 |  | - |  | - |
| HDL (mmol/l) | 1649 | 1.2 ± 0.3 | 1949 | 1.6 ± 0.4 | 930 | 1.0 ± 0.3 | 1604 | 1.3 ± 0.3 |
| Total cholesterol (mmol/l) | 1650 | 4.7 ± 1.1 | 1949 | 5.5 ± 1.0 | 960 | 5.3 ± 1.1 | 1622 | 5.3± 1.1 |
| Triglyceride (mmol/l) | 1671 | 1.7 ±1.1 | 1949 | 1.3 ± 0.7 | 953 | 2.3 ± 1.4 | 1615 | 1.5± 1.0 |

The data are presented as mean±SD. BMI: Body Mass Index. HbA_1c_: haemoglobin A_1c_ (glucose bound to haemoglobin). HDL: high density lipoprotein. T2D: type 2 diabetes
