# Supplementary material for: Common Variants in the Type 2 Diabetes KCNQ1 Gene Are Associated with Impairments in Insulin Secretion During Hyperglycaemic Glucose Clamp
Source: PLoS One. 2012 Mar 5;7(3):e32148. doi: 10.1371/journal.pone.0032148 (PMC3293880; doi:10.1371/journal.pone.0032148)
Supplement: Table S2 — Data on diabetic complications in type 2 diabetes patients from the DCS West-Friesland, the ZODIAC and the EPIC-NL studies. (DOC) [file pone.0032148.s002.doc]

**Supplementary table 2.** Data on diabetic complications in type 2 diabetes patients from the DCS West-Friesland, the ZODIAC and the EPIC-NL studies

| Study | T2D patients with macrovascular complications | T2D patients with ascertained nephropathy | T2D patients with ascertained neuropathy | T2D patients with ascertained retinopathy |
| --- | --- | --- | --- | --- |
| DCS West-Friesland | NA | NA | NA | 279 |
| ZODIAC | 284 | 355 | 247 | 90 |
| EPIC-NL | NA | 87 | 86 | 96 |
| Total | 284 | 442 | 333 | 465 |

NA – data not available
