# Supplementary material for: Common Variants in the Type 2 Diabetes KCNQ1 Gene Are Associated with Impairments in Insulin Secretion During Hyperglycaemic Glucose Clamp
Source: PLoS One. 2012 Mar 5;7(3):e32148. doi: 10.1371/journal.pone.0032148 (PMC3293880; doi:10.1371/journal.pone.0032148)
Supplement: Table S3 — Clinical characteristics of the individual hyperglycaemic clamp study samples. Data are represented as means ± SD or median (interquartile range). NTR = Netherlands Twin Register. (DOC) [file pone.0032148.s003.doc]

**Supplementary table 3.** Clinical characteristics of the individual hyperglycaemic clamp study samples

|  | **Hoorn** | **Utrecht** | **NTR** |
| --- | --- | --- | --- |
| n (NGT/IGT) | 138 (0/138) | 74 (63/11) | 123 (116/ 7) |
| Gender (M/F) | 66/72 | 17/57 | 58/65 |
| Age (y) | 60.5 ± 8.7 | 46.0 ± 6.4 | 31.5 ± 6.4 |
| BMI (kg/m^2^) | 28.1 ± 4.1 | 25.8 ± 3.6 | 24.2 ± 3.5 |
| Fasting plasma glucose (mmol/l) | 6.3 ± 0.7 | 4.6 ± 0.5 | 4.6 ± 0.5 |
| 2-hr plasma glucose (mmol/l) | 8.8 ± 1.7 | 5.5 ± 1.6 | 5.4 ± 1.2 |
| Fasting plasma insulin (pmol/l) | 62 (46-89) | 36 (24-54) | 35 (27-52) |
| First-phase insulin response (pmol/l) | 586 (374-892) | 852 (584-1169) | 805 (602-1168) |
| Second-phase insulin response (pmol/l) | 254 (171-352) | 260 (189-354) | 218 (164-362) |
| Insulin sensitivity index  (μmol/min/kg/pmol/l) | 0.109 (0.068-0.167) | 0.180 (0.122-0.281) | 0.221 (0.144-0.318) |
| Disposition index  (μmol/min/kg) | 66 (43-94) | 162 (93-224) | 173 (138-226) |

Data are represented as means ± SD or median (interquartile range). NTR=Netherlands Twin Register
