# Supplementary material for: Common Variants in the Type 2 Diabetes KCNQ1 Gene Are Associated with Impairments in Insulin Secretion During Hyperglycaemic Glucose Clamp
Source: PLoS One. 2012 Mar 5;7(3):e32148. doi: 10.1371/journal.pone.0032148 (PMC3293880; doi:10.1371/journal.pone.0032148)
Supplement: Table S4 — The characteristics of the Zwolle study population. Data are presented as mean ±SD or median with interquartile range for non-normally distributed data or %.* P<0.05, ** P<0.01, ***P<0.001, tested with Student's t-test or Mann-Whitney U as appropriate. (DOC) [file pone.0032148.s004.doc]

**Supplementary table 4.** The characteristics of the Zwolle study population

| Characteristics | Total number of patients | Deceased patients | Surviving patients |
| --- | --- | --- | --- |
|  | N=914 | N=358 | N=556 |
| Age (in years) | 67.9 (±11.3) | 75.3 (±8.5) | 63.1 (±10.2)*** |
| Sex (% women) | 57.8 | 57.6 | 58.1 |
| Diabetes duration (in years) | 5.0 (2 -10) | 6.0 (3-12) | 5.0 (2-9)*** |
| Smoking (%) | 18.1 | 14.0 | 21.0** |
| BMI (kg/m²) | 28.9 (±4.6) | 28.5 (±4.5) | 29.2 (±4.6)* |
| Systolic blood pressure (mmHg) | 152.5 (±25.1) | 154.7(±26.6) | 151.1 (±24.1)* |
| HbA1c (%) | 7.3 (±1.1) | 7.4 (±1.1) | 7.3 (±1.2) |
| eGFR (ml/min/1,73 m²) | 74.3 (±27.1) | 61.3 (±21.1) | 82.7 (±27.2)*** |
| Total cholesterol-HDL ratio | 5.0 (±1.4) | 4.9 (±1.5) | 5.0 (±1.4) |
| Albumin creatinine ratio | 1.9 (1.0-5.9) | 3.8 (1.5-11.5) | 1.5 (0.8-3.6)*** |
| Macrovascular complications (%) | 31.1 | 43.3 | 23.2*** |

Data are mean ±SD or median with interquartile range for non-normally distributed data or %.* P<0.05, ** P<0.01, ***P<0.001, tested with Student’s t-test or Mann-Whitney U as appropriate.
