# Supplementary material for: Common Variants in the Type 2 Diabetes KCNQ1 Gene Are Associated with Impairments in Insulin Secretion During Hyperglycaemic Glucose Clamp
Source: PLoS One. 2012 Mar 5;7(3):e32148. doi: 10.1371/journal.pone.0032148 (PMC3293880; doi:10.1371/journal.pone.0032148)
Supplement: Table S5 — Effect of KCNQ1 variants rs151290, rs2237892 and rs2237895 on beta-cell function as assessed with hyperglycaemic clamp. Analysis was adjusted for glucose tolerance status (NGT/IGT), study center, age, gender and BMI. All variables were log-transformed before analysis. p-values were computed for different additive models using linear generalized estimating equations (GEE) which takes into account the family relatedness when computing the standard errors. Alleles in bold are the risk alleles for type 2 diabetes identified by previous studies. DI, disposition index; IGT, impaired glucose tolerance; ISI, insulin sensitivity index; ND, not determined; NGT, normal glucose tolerance. (DOC) [file pone.0032148.s005.doc]

**Supplementary table 5.** Effect of *KCNQ1* variants rs151290, rs2237892 and rs2237895 on beta-cell function as assessed with hyperglycaemic clamp.

**Hoorn study**

| SNP | Genotype (N) |  | 1st phase insulin response (pmol/l) | 2nd phase insulin response (pmol/l) | ISI (µmol • min^-1^ • kg^-1^ • pmol/l^-1^) | DI (µmol • min^-1^ • kg^-1^) |
| --- | --- | --- | --- | --- | --- | --- |
| **rs151290** |  |  |  |  |  |  |
|  | AA (11) |  |  |  |  |  |
|  | **C**A (36) | β (sem)^a^ | -0.072 (0.032) | -0.026 (0.030) | +0.076 (0.034) | +0.002 (0.039) |
|  | **CC** (91) | *p-*value ^a^ | **0.024** | 0.39 | **0.028** | 0.96 |
| **rs2237892** |  |  |  |  |  |  |
|  | TT (0) |  |  |  |  |  |
|  | **C**T (11) | β (sem) ^a^ | +0.097 (0.054) | +0.181 (0.074) | -0.166 (0.080) | -0.038 (0.071) |
|  | **CC** (123) | *p-*value ^a^ | 0.075 | **0.014** | **0.037** | 0.60 |
| **rs2237895** |  |  |  |  |  |  |
|  | AA (32) |  |  |  |  |  |
|  | A**C** (74) | β (sem) ^a^ | -0.033 (0.031) | +0.018 (0.032) | +0.038 (0.037) | +0.016 (0.036) |
|  | **CC** (31) | *p-*value ^a^ | 0.29 | 0.56 | **0.31** | 0.65 |

Utrecht and NTR studies combined

| SNP | Genotype (N) |  | 1st phase insulin response (pmol/l) | 2nd phase insulin response (pmol/l) | ISI (µmol • min^-1^ • kg^-1^ • pmol/l^-1^) | DI (µmol • min^-1^ • kg^-1^) |
| --- | --- | --- | --- | --- | --- | --- |
| **rs151290** |  |  |  |  |  |  |
|  | AA (8) |  |  |  |  |  |
|  | **C**A (54) | β (sem)^a^ | -0.028 (0.026) | -0.042 (0.029) | +0.043 (0.026) | +0.014 (0.024) |
|  | **CC** (135) | *p-*value ^a^ | 0.28 | 0.15 | 0.094 | 0.56 |
| **rs2237892** |  |  |  |  |  |  |
|  | TT (0) |  |  |  |  |  |
|  | **C**T (17) | β (sem) ^a^ | +0.026 (0.047) | +0.109 (0.045) | -0.056 (0.050) | -0.027 (0.048) |
|  | **CC** (178) | *p-*value ^a^ | 0.57 | **0.017** | **0.27** | 0.57 |
| **rs2237895** |  |  |  |  |  |  |
|  | AA (54) |  |  |  |  |  |
|  | A**C** (106) | β (sem) ^a^ | -0.021 (0.029) | -0.069 (0.029) | +0.060 (0.028) | +0.035 (0.020) |
|  | **CC** (32) | *p-*value ^a^ | 0.47 | 0.018 | **0.034** | 0.084 |

^a^ Adjusted for glucose tolerance status (NGT/IGT), study center, age, gender and BMI.

All variables were log-transformed before analysis. *p*-values were computed for different additive models using linear generalized estimating equations (GEE) which takes into account the family relatedness when computing the standard errors. Alleles in bold are the risk alleles for type 2 diabetes identified by previous studies.

DI, disposition index; IGT, impaired glucose tolerance; ISI, insulin sensitivity index; ND, not determined; NGT, normal glucose tolerance
